# 

**Published:** 1908-05-16

**Authors:** 


					TTTTTTTTTT
-URGLON GENERAL'S OFFICE
MAY-2b. 19C3
T.i.?r.ph,o Add?.,, THE MODERN
Osoedale, London. | MEDICAL JOURNAL. ?
N?w SERIES. No. 61, Vol. III. SatttrDAY
? No. 1133. Vol. XLIV. oATUKDAi,
May 16th, 1908.
PRICE THREEPENCE.

				

